# Lower serum creatinine to cystatin C ratio associated with increased incidence of frailty in community-dwelling elderly men but not in elderly women

**DOI:** 10.1007/s40520-024-02787-7

**Published:** 2024-07-04

**Authors:** Shixian Zhou, Peixia Wang, Linlin Sun, Xinxiu Zhao, Caixia Gong, Yichen Yang, Wen Ren, Yunmei Yang, Qin Zhang, JingJin Jiang

**Affiliations:** 1 Department of Geriatrics, The First Affiliated Hospital, School of Medicine, Zhejiang University, Zhejiang, Hangzhou, 310003 China; 2 Key Laboratory of Diagnosis and Treatment of Aging and Physic-Chemical Injury Diseases of Zhejiang Province, The First Affiliated Hospital, School of Medicine, Zhejiang University, Zhejiang, Hangzhou, 310012 China; 3 Department of General Practice, The First Affiliated Hospital, School of Medicine, Zhejiang University, Zhejiang, Hangzhou, 310003 China

**Keywords:** Serum creatinine, Cystatin C, Frailty, Sarcopenia, Elders

## Abstract

**Background:**

Sarcopenia has been reported to play an important role in frailty syndrome. The serum creatinine/serum cystatin C ratio (Scr/Cys C ratio) has recently been recognized as a valuable indicator for assessing sarcopenia. However, few studies have examined the association between serum creatinine/serum cystatin C ratio and frailty. The objective of this study is to investigate the relationship between the serum creatinine/serum cystatin C ratio and frailty among older adults residing in the community.

**Methods and materials:**

A Total of 1926 community-dwelling older adults aged ≥ 60 years in the 2011 waves of the China Health and Retirement Longitudinal Study (CHARLS) were included. The participants’ frailty status was determined using a 39 item frailty index (FI), which classified individuals as “robust” (FI ≤ 0.1), “pre-frailty” (0.1 < FI < 0.25), or “frailty” (FI ≥ 0.25). The Scr/Cys C ratio was determined by dividing the serum creatinine level (mg/dL) by the cystatin C level (mg/L). The one-way analysis of variance(ANOVA) and Chi-squared test (χ2)were applied to compare the differences between the 3 groups. Both linear regression and logistic regression models were used to further investigate the relationship between Scr/Cys C ratio and frailty.

**Results:**

After adjusting for potential confounding factors, the study revealed that participants in the Q1 quartile of Scr/Cys C ratio had increased odds of frailty (Q1vs.Q4: OR = 1.880, 95% CI 1.126–3.139, p = 0.016) compared with those in the Q4 quartile group. In fully adjusted logistic regression models, male participants in the Q2 quartile of Scr/Cys C ratio were significantly correlated with higher odds of pre-frailty (Q2 vs.Q4: OR = 1.693, 95%CI 1.040–2.758, p = 0.034). However, this correlation was not observed in females (OR = 0.984, 95% CI 0.589–1.642, p = 0.950,). Additionally, the study observed an increase in both the frailty index and the incidence of frailty as age increased in both males and females.

**Conclusion:**

Among community-dwelling older adults, lower Serum creatinine to cystatin C ratio were found to be associated with increased odds of frailty prevalence in males.

**Supplementary Information:**

The online version contains supplementary material available at 10.1007/s40520-024-02787-7.

## Introduction

Over the past few decades, there has been a significant increase in the elderly population, both in terms of absolute numbers and relative proportions. Demographic projections indicate that this trend of population aging will continue to persist and even intensify over the next three decades^1^. The elderly over 80 years old will become the fastest growing group among the elderly population, which is expected to reach about 100 million people by 2050 in China. This demographic phenomenon plays a crucial role in the rising prevalence of frailty within society.

Frailty is a medical condition characterized by a decline in strength, endurance, and physiological function, leading to an increased vulnerability of individuals to developing heightened dependency and/or mortality^2^. The elderly population is at risk of frailty, and the prevalence of frailty in those aged 80 and older is significantly higher than that in the lower age population. Frailty has been shown to be correlated with higher rates of mortality, hospitalization, falls, fractures, and physical disabilities in elderly populations [3, 4]. The frailty index, which assesses frailty, is considered an emerging indicator of biological age [5–8]. It includes the accumulation of health-related defects, such as signs, symptoms, illness, and disability. Serum creatinine and cystatin C are widely used biomarkers in clinical practice to evaluate kidney function [9]. Serum creatinine varies with body composition while cystatin C is widely found in various types of nucleated cells and is relatively less affected by muscle mass, sex, age, or nutritional status [10, 11]. Kashani et al. utilized the serum creatinine/serum cystatin C ratio (called by them sarcopenia index or SI) for the estimation of sarcopenia 12. SI is considered a reliable biomarker of skeletal muscle mass. The relationship between SI and depression or cognitive function have been previously investigated [13], however, it remains unclear whether a lower serum creatinine/serum cystatin C ratio is associated with an increased incidence of frailty. In our study, we aimed to examine the relationship between serum creatinine/serum cystatin C ratio and frailty and the predictive value of serum creatinine/serum cystatin C ratio in estimating frailty in community-dwelling Chinese old adults.

## Methods

### Study population

This study implemented cross-sectional analysis design using data from the 2011 China health and retirement longitudinal study (CHARLS). CHARLS is a nationally representative cohort comprising community-dwelling adults aged 45 years and older in China. The study is specifically designed to gather comprehensive information on various aspects, including demographic background, family information, family care and insurance, health status and functioning, household income, and individual characteristics [14]. For the baseline survey of CHARLS, a total of 17,708 participants were randomly selected from 450 urban communities and rural areas across 150 counties and districts in 28 provinces in China. The survey was conducted between June 2011 and March 2012. Subsequently, these participants were followed up every 2 years. Among the 17,708 participants, 13,778 participants attended the anthropometric and physical function measurements to collect data of height, weight, waist circumference, blood pressure, grip strength, gait speed, and other physical function indicators. 11,847 participants provided the blood samples for laboratory tests. CHARLS has received approval from the Research Ethics Committee of Peking University (IRB00001052–11015). Informed consent was obtained from all participants or their legal representatives, ensuring ethical considerations were upheld. For more detailed information about CHARLS, you can visit the project website at http://charls.pku.edu.cn/.

**Fig. 1:**
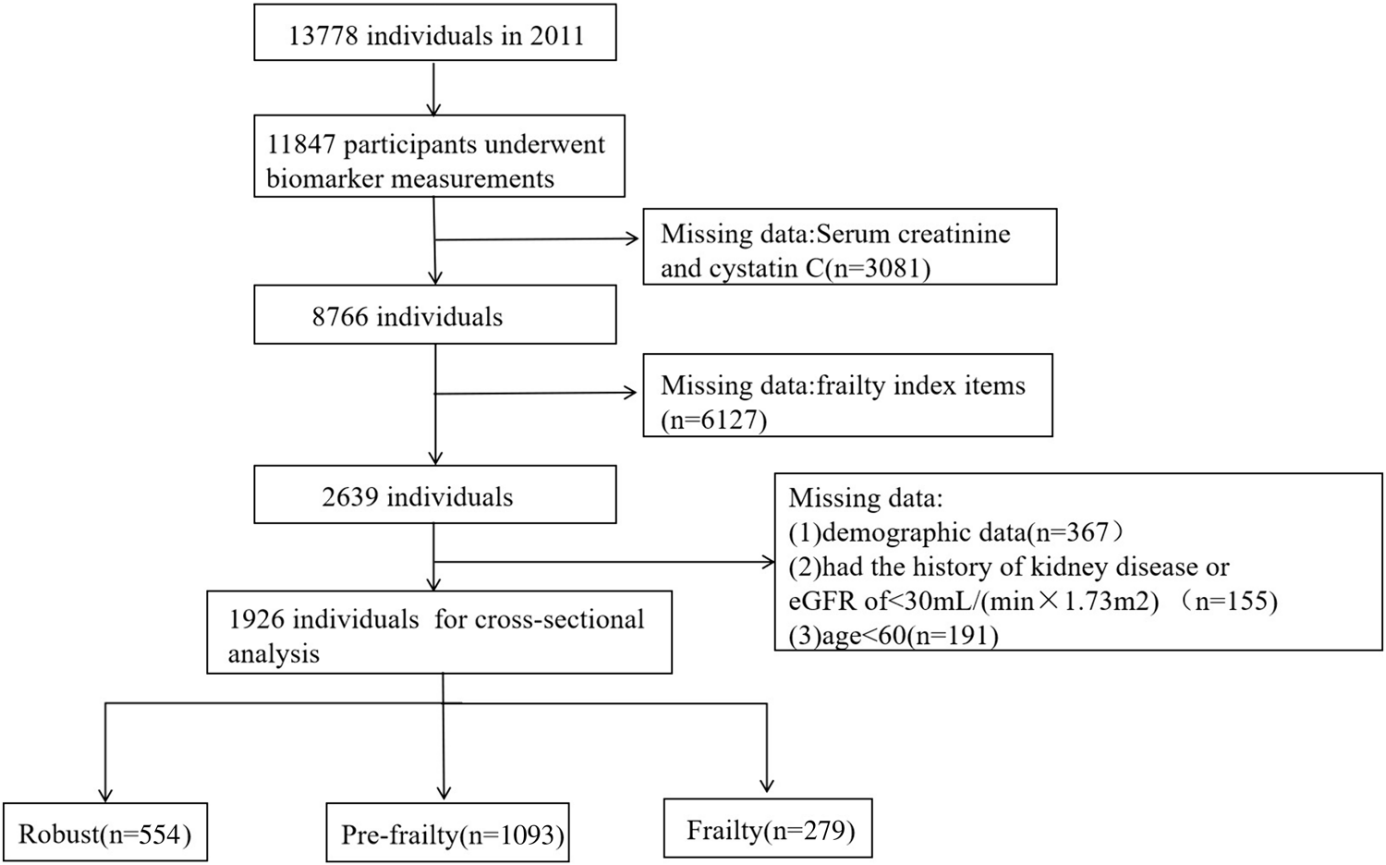
Flowchart of the study identifcation process

For this cross-sectional analysis, participants who do not meet the following criteria will be excluded: (1)missing serum creatinine and cystatin C(n = 3081); (2) missing frailty index items(n = 6127); (3)missing demographic data( (n = 367); (4)individuals aged < 60 years old (n = 191); (5) had the history of kidney disease or an estimated glomerular filtration rate (eGFR) < 30 mL/(min × 1.73m^2^) by using the Chinese-based equation(n = 155) [15]. Finally, a total of 1926 participants were included in the analysis. The selection process of individuals included in the study is shown in Fig. 1.

### Data collection

The demographic characteristics of the participants in this study include age, sex, educational level, marital status, residence status, smoking and alcohol drinking history, as well as a history of chronic diseases (including hypertension, diabetes or high blood sugar, heart disease, stroke, cancer, chronic lung disease, hepatic disease, stomach or other digestive disease, arthritis or rheumatism, dyslipidemia, emotional, nervous, or psychiatric problems, memory-related diseases and asthma) were obtained from the questionnaires.

In this study, age was considered as a continuous variable. Sex was treated as a binary variable with two categories: male or female. Educational level was divided into no formal education (illiterate), elementary school or below(including did not finish primary school but capable of reading or writing, sishu/home school, and elementary school), middle school (including middle school, high school and vocational school), College or above (including 2–3 Year College/Associate degree, 4 Year College /Bachelor’s degree, Master’s degree and Doctoral degree/Ph.D.). Marital status was classified into married and unmarried (including separated, divorced, widowed, and never married). Smoking and alcohol drinking history were collected through self-reporting, and participants were asked to answer “yes” or “no” to indicate whether they had a history of smoking or alcohol consumption. These variables were treated as binary variables. The history of chronic diseases were collected by using questions such as “Have you been diagnosed with [conditions listed below, read one by one] by a doctor?”.

### Anthropometric assessment

Each participant underwent a physical exam, including measurements of height, weight, and waist circumference. Shoes were removed for height measurements using a meter. Then, shoes, heavy coats, and weights were removed for body weight measurements on a scale. Standing up without the heavy coat, the interviewer measured waist circumference at the navel. BMI was calculated as weight in kg divided by height squared in meters.

### Serum creatinine and cystatin C

In the study, blood samples were collected from each participant by trained staff members from the Chinese Center for Disease Control and Prevention (Chinese CDC). A total of 11,847 participants underwent biomarker measurements including C reflects protein (CRP), glycated hemoglobin (HbA1c), fasting blood glucose (FBG), total cholesterol (TC), triglycerides (TG), low-density lipoprotein cholesterol (LDL-c), high-density lipoprotein cholesterol (HDL-c), hemoglobin, serum cystatin C, serum creatinine, urea nitrogen and uric acid. Serum creatinine and cystatin C measurements were conducted using the rate-blanked and compensated Jaffe creatinine method and the particle-enhanced turbidimetric assay method, respectively.

### Frailty index

The frailty index was implemented using a standardized procedure, which closely resembled the protocols employed in previous studies conducted by CHARLS [16].The frailty index items included activity of daily living (ADL) (6 items), instrumental activity of daily living (IADL) (5 items), physical function limitations (9 items), chronic diseases (12 items), mental health indicators (5 items), subjective functioning (self-rated health) and BMI. Each health deficit was assigned a value ranging from 0.00 (no deficit) to 1.00 (all items exhibiting deficits) on an interval scale (for more details, refer to Supplementary Table S1). We included participants with a total of 39 health deficits. Following established methodologies, we calculated the frailty index by summing all health deficits and dividing it by the total number of health deficits. We categorized the resulting continuous frailty index into three groups: robust (frailty index ≤ 0.10), pre-frailty (frailty index > 0.10 to < 0.25), and frailty (frailty index ≥ 0.25) [17].

### Statistical analysis

Scr/Cys C ratio was divided into quartiles and treated as categorical variables. Participants were grouped as ‘Q1 < 0.624’, ‘0.624 ≤ Q2 < 0.718’, ‘0.718 ≤ Q3 < 0.830,’ and ‘Q4 ≥ 0.830’. Categorical variables were presented using frequencies (percentages), while continuous variables were presented using means and standard deviations (SD) or medians (interquartile range, IQR). Continuous variables between the robust, pre-frailty, and frailty groups were compared using one-way analysis of variance (ANOVA), and Bonferroni correction was applied for multiple comparisons. Meanwhile, ANOVA was also utilized to compare differences in the frailty index among groups divided based on quartiles of the Scr/Cys C ratio. T-tests were employed to compare differences in the frailty index between different genders within the same age group. Categorical variables among the four groups divided by Scr/Cys C ratio quartiles were compared using the nonparametric tests, while other disordered categorical variables among groups were compared using Chi-squared test.

Unadjusted and adjusted linear regression models were applied to explore the correlation between the Scr/Cys C ratio and the frailty index, with the Scr/Cys C ratio serving as the independent variable and the frailty index as the dependent variable. Unadjusted and adjusted logistic regression models were used to assess the impact of the Scr/Cys C ratio on the risk of developing frailty, where the Scr/Cys C ratio quartiles were treated as the independent variables and the outcomes (Robust, Pre-frailty, and Frailty) as the dependent variable. In the unadjusted models (Model 1), no confounders were controlled for. In the adjusted models (Model 2), potential confounding factors, including age, sex, education level, marital status, history of smoking, history of alcohol consumption, residence status, BMI, waist circumference, and chronic diseases (including hypertension, diabetes, heart disease, stroke, arthropathy or rheumatoid disease, and dyslipidemia), were adjusted. Odds ratios (ORs) and their corresponding 95% confidence intervals (CIs) were calculated from the logistic regression models. A p-value of less than 0.05 (2-sided) was considered statistically significant. IBM SPSS Statistics 26 was used for data interpretation and analysis.

## Results

### Baseline characteristics of participants

**Fig. 2:**
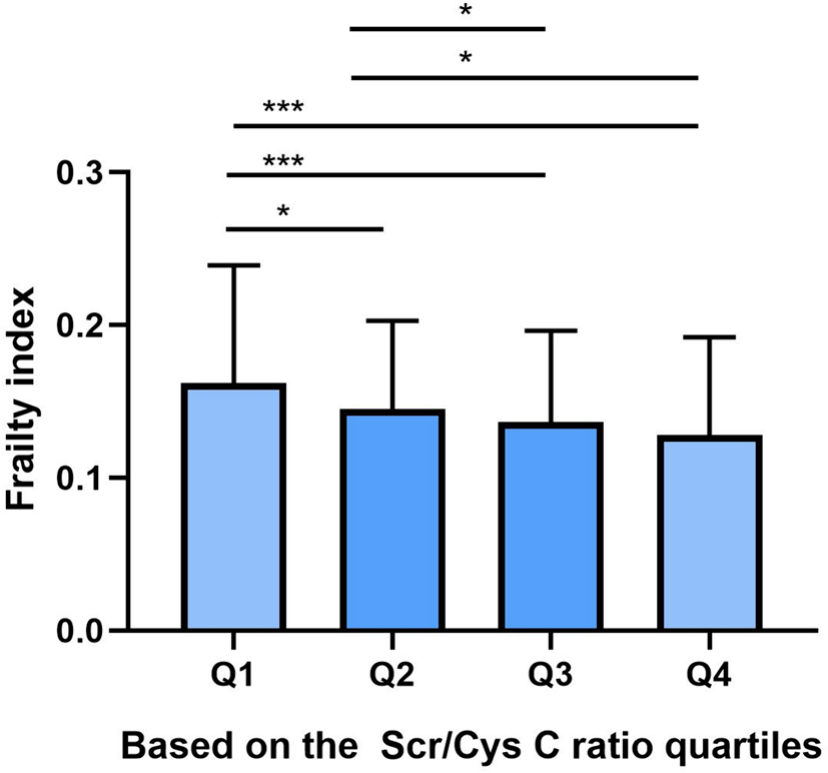
The differences of frailty index among four groups according to Scr/Cys C ratio quartiles, *p < 0.05, ***p < 0.001

A total of 1926 participants were enrolled in our study, 856 (44.4%) of them were males. We further divided the participants into four groups by using the quartiles of the Scr/Cys C ratio as cut-points: Q1 (Scr/Cys C ratio < 0.624, n = 481), Q2 (0.624 ≤ Scr/Cys C ratio < 0.718, n = 483), Q3 (0.718 ≤ Scr/Cys C ratio < 0.830, n = 482) and Q4 (Scr/Cys C ratio ≥ 0.830, n = 480). As shown in Table 1, the results showed that sex, age, BMI, waist circumference, history of smoking, history of drinking, residence status, education level, marital status, and chronic diseases (including hypertension, diabetes, stroke, arthropathy or rheumatoid disease, and dyslipidemia) were significantly different among the four groups. Additionally, the results showed that the number of men, BMI, history of smoking, history of drinking (including those who drink less than once a month and those who drink more than once a month), lived in urban, had educational experience (including elementary school, middle school and college or above), married, the prevalence of diabetes and serum creatinine increased with the higher Scr/Cys C ratio. Conversely, the age, no history of drinking, lived in rural area, no formal education illiterate, serum cystatin C, eGFR and frailty index decreased with the higher Scr/Cys C ratio levels. It is worth noting that the frailty index decreases as the Scr/Cys C ratio increase(Fig. 2).

**Table 1:** Characteristics of participants according to Scr/Cys C ratio quartiles

Characteristics	Total	Quartiles of Scr/Cys C ratio				P value
Number of people	n = 1926	Q1(n = 481)	Q2(n = 483)	Q3(n = 482)	Q4(n = 480)	
Male, n (%)	856(44.44)	117(24.32)	175(36.23)	253(52.49)	311(64.79)	< 0.001
Age(year), mean ± SD	69.41 ± 6.92	72.12 ± 7.30	69.79 ± 7.06	68.41 ± 6.28	67.31 ± 6.02	< 0.001
BMI	23.12 ± 4.19	22.56 ± 4.08	22.65 ± 4.16	23.32 + 3.91	23.94 ± 4.44	< 0.001
Waist circumference(cm)	85.11 ± 13.21	84.65 ± 13.77	84.00 ± 13.06	85.25 ± 13.65	86.56 ± 12.23	0.020
History of smoking, n (%)	774(40.19)	143(29.73)	182(37.68)	208(43.15)	241(50.21)	< 0.001
History of drinking, n (%)						0.001
None of these	1423(73.88)	387(80.46)	366(75.78)	342(70.95)	328(68.33)	
Drink but less than once a month	90(4.67)	17(3.53)	19(3.93)	24(4.98)	30(6.25)	
Drink more than once a month	413(21.44)	77(16.01)	98(20.29)	116(24.07)	122(25.42)	
Residence status, n (%)						< 0.001
rural	1564(81.20)	417(86.69)	414(85.71)	387(80.29)	346(72.08)	
Urban	362(18.80)	64(13,31)	69(14.29)	95(19.71)	134(27.92)	
Education, n (%)						< 0.001
No formal education illiterate	814(42.26)	280(58.21)	226(46.79)	173(35.89)	134(27.92)	
Elementary school	831(43.15)	165(34.30)	204(42.32)	219(45.43)	243(50.63)	
Middle school	258(13.40)	35(7.28)	50(10.35)	82(17.01)	91(18.96)	
College or above	23(1.19)	1(0.21)	3(0.62)	7(1.45)	12(2.50)	
Married, n (%)	1494(77.57)	314(65.28)	373(77.23)	400(82.99)	407(84.79)	< 0.001
Chronic disease, n (%)						
Hypertention	679(35.30)	149(31.00)	180(37.30)	156(32.40)	194(40.40)	0.007
Diabetes	137(7.11)	24(4.99)	28(5.80)	30(6.22)	55(11.46)	< 0.001
Heart disease	348(18.07)	79(16.42)	92(19.05)	74(15.35)	103(21.46)	0.062
stroke	74(3.84)	23(4.78)	8(1.66)	18(3.73)	24(5.00)	0.026
Chronic lung disease	282(14.64)	66(13.72)	67(13.87)	82(17.01)	67(13.96)	0.407
Cancer	14(0.73)	2(0.42)	4(0.83)	4(0.83)	4(0.83)	0.835
Hepatic disease	73(3.79)	10(2.08)	25(5.18)	16(3.32)	22(4.58)	0.057
Stomach or other Digestive system disease	438(22.74)	109(22.66)	121(25.05)	101(20.95)	107(22.29)	0.494
Arthropathy or rheumatoid disease	747(38.79)	204(42.41)	199(41.20)	162(33.61)	182(37.92)	0.024
Dyslipidemia	230(11.94)	47(9.77)	46(9.52)	53(11.00)	84(17.50)	< 0.001
Emotional tension or mental problems	37(1.92)	10(2.08)	10(2.07)	9(1.87)	8(1.67)	0.961
Memory-related diseases	31(1.61)	10(2.08)	7(1.45)	6(1.24)	8(1.67)	0.762
Asthma	115(5.97)	26(5.41)	24(4.97)	35(7.26)	30(6.25)	0.452
Laboratory data, mean ± SD						
Serum creatinine (mg/dL)	0.80 ± 0.20	0.69 ± 0.18	0.78 ± 0.17	0.84 ± 0.19	0.91 ± 0.20	< 0.001
Serum cystatin C(mg/L)	1.12 ± 0.29	1.27 ± 0.33	1.16 ± 0.25	1.08 ± 0.25	0.95 ± 0.22	< 0.001
eGFR(ml/(min × 1.73m2)	74.70 ± 9.18	78.26 ± 10.87	74.37 ± 8.50	74.06 ± 8.4	72.09 ± 7.46	< 0.001
Frailty index (IQR)	0.14(0.11)	0.16(0.14)	0.15(0.10)	0.14(0.11)	0.13(0.11)	< 0.001

### Incidence of frailty

**Fig. 3:**
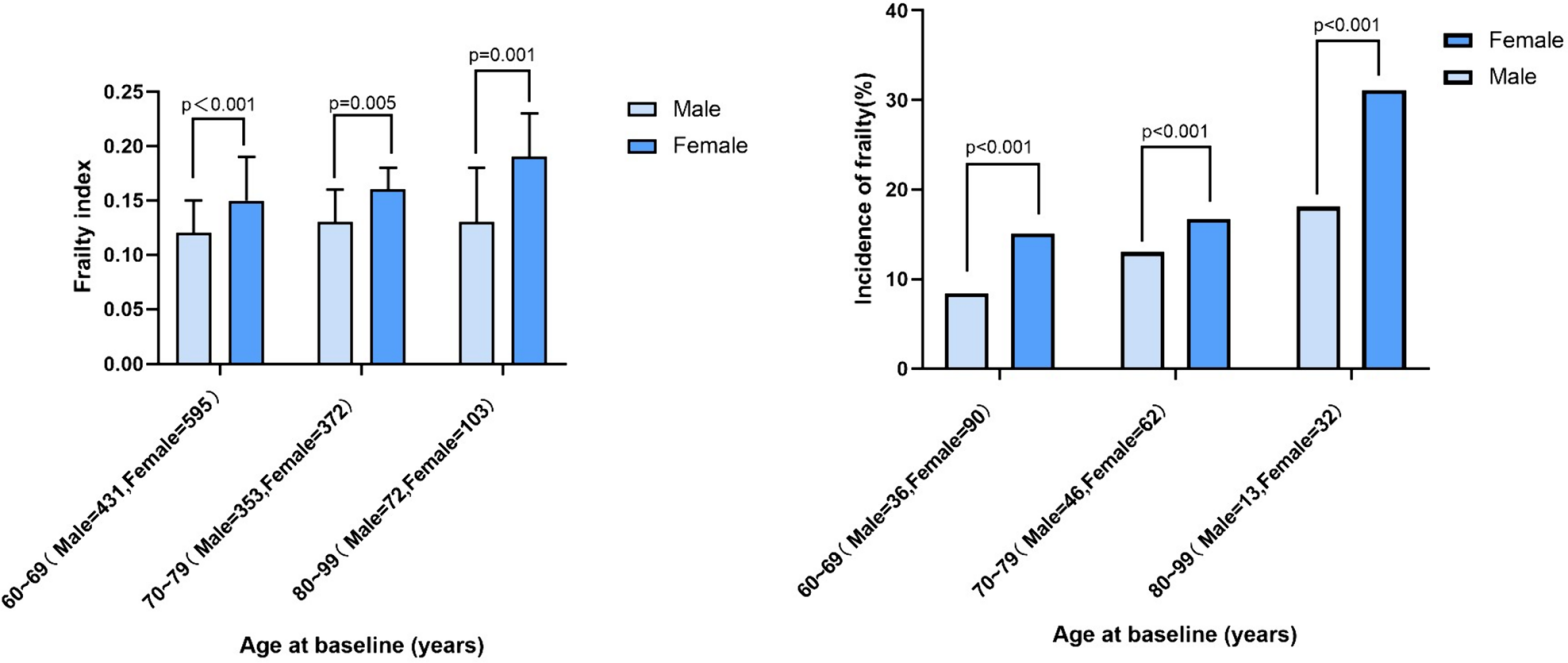
The frailty index and incidence of frailty according to age, stratified by gender

We further divided these participants into three groups according to age and stratified analyses by sex. Due to the small number of individuals over the age of 90, we have divided the ages into three groups: 60 ~ 69 years, 70 ~ 79 years, and 80 ~ 99 years.We found that the frailty index and the incidence of frailty (the proportion of participants with a frailty index ≥ 0.25) increased with age in both males and females. Furthermore, it was discovered that males exhibited lower frailty index and a lower incidence of frailty compared to females (Fig. 3).

### Association between the Scr/Cys C ratio and frailty

To further investigate the relationship between Scr/Cys C ratio and frailty index, linear regression models were performed (Table 2). Model 1 suggests a significant negative correlation between frailty and the Scr/Cys C ratio in the total population(β = −0.037, 95 CI% −0.059–−0.014, p = 0.002), a correlation that does not hold when the data is stratified by gender. In contrast, Model 2 reveals no significant linear correlation across any of the groups examined. To shed more light on the association between the Scr/Cys C ratio and frailty, we performed a comprehensive logistic regression analysis, the results of which were detailed in Table 3. The Scr/Cys C ratio was treated as a categorical variable based on the quartile cut-off points. Using the Q4 quartile as the reference group, unadjusted models revealed that the Q1quartile of Scr/Cys C ratio was significantly correlated with a higher odds of frailty (Q1 vs. Q4: OR: 2.305, 95% CI 1.547–3.434, p < 0.001). After adjusting for multiple confounding factors in model 2, the observed correlation between the Q1 quartile of Scr/Cys C ratio and frailty remained significant (Q1 vs. Q4: OR: 1.880, 95% CI 1.126–3.139, p = 0.016). In addition, we did not observed the similar relationship between the Scr/Cys C ratio and prefrailty.

**Table 2:** Linear regression models for correlations between frality index and Scr/Cys C ratio as a continuous predictor

Outcome	Model 1		Model 2	
	β(95% CI)	p	β(95% CI)	p
Total population	−0.037 (−0.059, -0.014)	0.002	−0.011 (−0.032, 0.010)	0.292
Female	−0.016 (−0.049, 0.017)	0.336	−0.003 (−0.032, −0.027)	0.863
Male	−0.021 (−0.054, 0.011)	0.200	−0.019 (−0.049, 0.011)	0.212

Model 1: unadjusted model Model 2: adjusted for age, sex(total population), education level, marital status, history of smoking, history of alcohol drinking status, residence status, BMI, waist circumference and chronic diseases (including hypertension, diabetes, heart disease, stroke, arthropathy or rheumatoid disease and dyslipidemia)

**Table 3:** Logistic regression for frailty with Scr/Cys C ratio by quartiles; Odds Ratios [95% CIs]

Outcome	Model 1		Model 2	
	OR(95% CI)	p	OR(95% CI)	p
Robust (N = 554)	ref		ref	
Pre-frailty(N = 1093)				
Q1 vs.Q4	1.323(0.988, 1.773)	0.061	1.178(0.825, 1.683)	0.368
Q2 vs.Q4	1.430(1.072, 1.909)	0.015	1.293(0.921, 1.814)	0.137
Q3 vs.Q4	1.161(0.879, 1.534)	0.293	1.386(1.006, 1.911)	0.046
Frailty(N = 279)				
Q1 vs.Q4	2.305(1.547,3.434)	< 0.001	1.880(1.126, 3.139)	0.016
Q2 vs.Q4	1.609(1.062, 2.440)	0.025	1.463(0.882, 2.425)	0.140
Q3vs.Q4	0.808(0.517, 1.264)	0.351	1.046(0.620, 1.764)	0.866

Model 1: unadjusted model Model 2: adjusted for age, sex, education level, marital status, history of smoking, history of alcohol drinking status, residence status, BMI, waist circumference and chronic diseases (including hypertension, diabetes, heart disease, stroke, arthropathy or rheumatoid disease and dyslipidemia)

To account for gender influence on frailty, we conducted separate logistic regression analyses for males and females (Table 4). The significant negative relationship between the Scr/Cys C ratio and frailty or pre-frailty was only observed in the male population, not in the female population. In males, individuals in the Q1 quartile of the Scr/Cys C ratio was significantly correlated with higher odds of frailty in the unadjusted Model 1(Q1 vs. Q4: OR: 1.969, 95% CI 1.009–3.841, p = 0.047). But after adjusting for confounding factors in Model 2, the correlation between the Q1 quartile of Scr/Cys C ratio and frailty remained less pronounced (Q1 vs. Q4: OR:2.228, 95% CI 0.965–5.145, p = 0.061). More importantly, we also discovered that males in the Q2 quartile of the Scr/Cys C ratio exhibited significantly higher odds of pre-frailty in both the unadjusted model 1 (Q2 vs. Q4: OR: 1.569, 95% CI 1.037–2.374, p = 0.033) and adjusted Model 2 (Q2 vs. Q4: OR: 1.693, 95% CI 1.040–2.758, p = 0.034).

**Table 4:** Gender-stratified logistic regression for frailty with Scr/Cys C ratio by quartiles; Odds Ratios [95% CIs]

Female	Model 1		Model 2		Male	Model 1		Model 2	
	OR(95% CI)	p	OR(95% CI)	p		OR(95% CI)	p	OR(95% CI)	p
Robust	ref		ref			ref		ref	
Pre-frailty									
Q1 vs.Q4	1.017 (0.652, 1.589)	0.940	1.031 (0.615, 1.727)	0.909		1.099 (0.687, 1.759)	0.693	1.197 (0.689, 2.079)	0.524
Q2 vs.Q4	1.014 (0.645, 1.595)	0.952	0.984 (0.589, 1.642)	0.950		1.569 (1.037, 2.374)	0.033	1.693 (1.040, 2.758)	0.034
Q3 vs.Q4	1.046 (0.652, 1.680)	0.852	1.258 (0.737, 2.148)	0.401		1.097 (0.770, 1.583)	0.609	1.429 (0.941, 2.172)	0.094
Frailty									
Q1 vs.Q4	1.482 (0.838, 2.622)	0.176	1.460 (0.728, 2930)	0.286		1.969 (1.009, 3.841)	0.047	2.228 (0.965, 5.145)	0.061
Q2 vs.Q4	1.009 (0.555, 1.836)	0.976	0.946 (0.465, 1.923)	0.878		1.724 (0.909, 3.268)	0.095	2.065 (0.939, 4.540)	0.071
Q3vs.Q4	0.581 (0.294, 1.148)	0.118	0.625 (0.281, 1.391)	0.250		0.908 (0.493, 1.671)	0.757	1.384 (0.670, 2.862)	0.380

Model 1: unadjusted model Model 2: adjusted for age, education level, marital status, history of smoking, history of alcohol drinking status, residence status, BMI, waist circumference and chronic diseases (including hypertension, diabetes, heart disease, stroke, arthropathy or rheumatoid disease and dyslipidemia)

## Discussion

In this study, we conducted a thorough investigation into the correlation between the serum creatinine/cystatin C ratio (Scr/Cys C ratio) and the frailty index among older adults residing in the community of China. We discovered that the Q1 quartile of Scr/Cys C ratio was independently associated with a higher risk of frailty in logistic regression models after adjustment for confounding factors. When we analyzed the data separately by gender in logistic regression models, we found no significant correlation between the Scr/Cys C ratio and frailty risk in women. However, in men, a lower Scr/Cys C ratio, particularly in the Q2 quartile, was independently associated with a higher risk of pre-frailty after adjusting for confounding factors. Further studies are needed to explore the underlying reasons for the difference by gender.

Both serum creatinine (SCr)and cystatin C (CysC) are commonly used serum markers in clinical practice to assess the glomerular filtration rate (GFR) and evaluate kidney function. Creatinine is a metabolite of creatine phosphate metabolism primarily generated during muscle energy metabolism, and its serum concentration is influenced by the skeletal muscle content of the body. Based on the direct correlation between muscle mass and SCr levels, the latter has been used to indicate muscle mass [18]. Scr levels decrease in patients with sarcopenia. Cystatin C is a protein involved in intracellular protein catabolism that is produced at a constant rate by all nucleated body cells [19]. Cystatin C is primarily filtered through the glomeruli within the kidneys. Apart from conditions such as diabetes and metabolic syndrome, elevated levels of serum cystatin C may indicate alterations in glomerular filtration rate, as in conditions like Shrunken Pore Syndrome (SPS) [20, 21], where the glomerular filtration pore size is reduced, resulting in elevated levels of cystatin C in the blood. Compared to creatinine, cystatin C is less affected by a patient’s muscle mass, making it a more accurate indicator of kidney function in some cases. This makes cystatin C a very useful biomarker for assessing kidney health, particularly in elderly individuals or patients in disease states where muscle mass may vary significantly. Therefore, a high Scr/Cys C ratio not only reflects good muscle mass but may also be a positive indicator of glomerular filtration function in the kidneys. Scr/Cys C ratio (SI)has been suggested as a surrogate marker for sarcopenia 22–25and has been utilized as a surrogate marker for muscle mass in amyotrophic lateral sclerosis (ALS) [26], lung transplant candidate [27], and diabetes [28, 29]. Previous studies have shown that SI was inversely proportional to disease severity and significantly lower than in healthy controls in ALS patients. This was further investigated in a prospective study by Ren et al [30]. The SI demonstrated a robust association with long-term mortality, malnutrition, and sarcopenia in elderly Chinese patients [30–32]. Sarcopenia is associated with dysfunction, frailty, risk of death, cognitive dysfunction as well as other adverse outcomes. Sarcopenia and frailty syndrome are closely related, with sarcopenia being a significant component of the frailty syndrome. Both conditions are recognized as strong predictors of morbidity, disability, and mortality in the elderly population. This prompted us to examine the relationship between serum creatinine/serum cystatin C ratio and frailty index, as both are readily available clinically and can be widely used.

Gender differences in the Scr/Cys C ratio have been observed in several studies, likely due to variations in serum creatinine concentration between genders. Consequently, in addition to evaluating the entire population, we conducted separate analyses for each gender. Initially, using linear regression analysis, we found that the Scr/Cys C ratio and frailty index had a negative correlation in the unadjusted models, but this association disappeared after adjusting for confounding factors and considering gender differences, suggesting a potential interaction between gender and the Scr/Cys C ratio's effects on frailty. In the multivariate logistic regression analysis of the total population,the lowest(Q1) quartile of Scr/Cys C ratio was consistently linked to a higher odds of frailty after adjustments. Among males, we observed similar changes, although after adjusting for various confounding factors, this correlation became less pronounced. Consistent with this, we found that the frailty index decreased with an increase in the Scr/Cys C ratio. To explain the relationship between the Scr/Cys C ratio and frailty, we mainly attribute it to the effects of sarcopenia and frailty since Scr/Cys C ratio is regarded as a reliable biomarker for sarcopenia. Studies have shown that sarcopenia is a key component of physical frailty, and both interact and form a vicious cycle. In the study conducted by Meerkerk et al., it was discovered that the prevalence of sarcopenia was significantly higher among frailty patients compared to individuals who were not debilitated. This suggests a strong association between sarcopenia and frailty, indicating that sarcopenia is more common in individuals with frailty. In addition, it was found that patients with sarcopenia are more likely to frailty. Other researchers have found that individuals who experience debilitation or have functional limitations are more prone to developing sarcopenia. This suggests that sarcopenia can be considered a risk factor for frailty. However, the causal relationship between the two is still unclear at the present stage, and large-scale longitudinal studies need to be discussed in-depth.

Additionally, males in the Q2 quartile of the Scr/Cys C ratio exhibited significantly higher odds of pre-frailty in both the unadjusted and adjusted models. As we know, pre-frailty is the transitional stage in which the elderly move from a state of health to one of overt frailty. In this phase, individuals may start to experience early symptoms of frailty, such as a slight decline in physical strength, reduced activity levels, or unexplained weight loss. These findings suggest that in males, the risk of frailty may start to rise when the Scr/Cys C ratio is in the Q2 quartile. By the time individuals reach the Q1 quartile of the Scr/Cys C ratio, they might already be in a frailty state. These research findings are significant as they provide insights into the early detection of elderly individuals in the pre-frailty stage. However, further research is needed to confirm these findings definitively.This allows for timely preventive measures to be taken, which can help stop or slow down further decline. For females, although an increased risk of frailty was observed in the lowest quartile, this trend was not statistically significant, indicating different impacts of the Scr/Cys C ratio on frailty between genders. At present, the underlying reasons for this gender disparity remain unclear, necessitating further research for a comprehensive understanding in the future. Furthermore, we found that the frailty index and the incidence of frailty increased with age in both males and females. The incidence of frailty was found to be higher in females than in males among individuals. The finding is consistent with a previous study conducted in South Korea, which also reported a higher prevalence of physical frailty in women compared to men.

This study prompted us to focus more on community-dwelling older adults or older patients with a low Scr/Cys C ratio. Early preventive action is essential to reduce the occurrence of frailty and to mitigate the adverse consequences associated with frailty. However, there were still some limitations that should be noticed. First, all the health information about the study subjects was collected by questionnaire, which may introduce information bias in the self-report of the respondents. For example, the diagnosis of chronic diseases was based on self-reporting, and some participants may be unaware that they have a disease. Second, This study is a cross-sectional study design, so particular caution should be taken in interpreting the findings. As this research approach may be difficult to determine the exact causal relationship between frailty and their influencing factors. Besides, cognitive function was not included in the construction of the frailty index because of missing data, which may have caused bias in estimates of frailty prevalence that may be inconsistent with the results reported in previous studies. Finally, excluding participants with incomplete data may introduce some selection bias. All the data used in this study were obtained from CHARLS, which is a representative national database of middle-aged and older adults in China. However, it is unclear whether this conclusion can be extrapolated to other countries.

## Conclusions

In summary, our study investigated the correlation between the Scr/Cys C ratio and frailty in an older Chinese population using the CHARLS database. Our findings unveiled a significant correlation between the Q1 quartile of the Scr/Cys C ratio and increased odds of frailty in the older Chinese population. However, when analyzing by gender separately, we only observed that male individuals in the Q1 quartile of the Scr/Cys C ratio were significantly correlated with higher odds of frailty in the unadjusted model. Additionally, the Q2 quartile of the Scr/Cys C ratio and pre-frailty were significantly negatively correlated in both unadjusted and adjusted models. In contrast, among females, we did not observe any correlation between the Scr/Cys C ratio and pre-frailty or frailty. Further large-scale population studies may be needed to verify the impact of Scr/Cys C ratio on frailty in different genders.

### Supplementary Information

Below is the link to the electronic supplementary material.

Supplementary file1 (XLSX 590 KB)

## Data Availability

The authors confirm that the data supporting the findings of this study are available within the article and its supplementary materials.
